# A qualitative exploration of the experiences of adults with cystic fibrosis unable to benefit from triple combination therapy

**DOI:** 10.1177/13591053251369393

**Published:** 2025-10-02

**Authors:** Mulongwe Mwelwa, Christopher Hobson, Steven Stirk, Heledd Lewis, Victoria Samuel

**Affiliations:** 1 Cardiff University, UK; 2 Cardiff and Vale University Health Board, Heath Park, UK

**Keywords:** Cystic fibrosis, ETI, Trikafta, Kaftrio, triple combination therapy, interpretative phenomenological analysis

## Abstract

Elexacaftor/Tezacaftor/Ivacaftor (ETI) is a highly effective modulator therapy that has transformed the lives of many people with Cystic Fibrosis (CF). However, limited research has explored the experiences of those not able to benefit from ETI due to genetic factors. Seven adults with CF participated in semi-structured interviews, and data were analysed using Interpretative Phenomenological Analysis (IPA). Four themes emerged: ‘feeling forgotten’, (the challenges of being in a minority group); ‘conflicted emotions’, (the emotional impact of comparing one’s situation with others); ‘fragility of hope’, (the journey of hope experienced by participants); and ‘remaining on the old CF trajectory’, (the ongoing challenges of living with CF). The findings suggest a need for improved communication, psychological and peer support, inclusive media representation and opportunities for active involvement in research and advocacy. Implementing these interventions may help this group feel seen and supported, even as the CF landscape continues to evolve.

## Introduction

### Cystic fibrosis and advancements in treatment

Cystic Fibrosis (CF) is a genetic condition that is caused by a mutation in the gene encoding the cystic fibrosis transmembrane conductance regulator (CFTR) protein. Malfunctioning CFTR protein disrupts the movement of salt and water in various organs leading to a build-up of thick and sticky mucus in areas such as the lungs, intestines and pancreas (Hine et al., 2022; Hisert et al., 2023).

CFTR mutations can cause diverse health issues, such as persistent coughing, recurring chest infections, pancreatic insufficiency in some patients, difficulties gaining weight and other related conditions such as bone diseases, CF-related diabetes, male infertility and liver problems (Bell et al., 2020; Chen et al., 2021). Managing CF requires a complex treatment regimen involving airway clearance methods, long term nebulised antibiotics and mucolytics, pancreatic enzymes, intravenous antibiotics and oral antibiotics over extended periods of time (Davies et al., 2020; Goldbeck et al., 2014). As a result, studies have indicated that anxiety and depression are 2–3 times higher amongst people with CF compared to the general population (Quittner et al., 2016).

Previously, available treatments for CF primarily addressed the symptoms and related complications associated with CF, rather than targeting specific genes but more recently the development of CFTR modulator therapies have meant that the genetic causes of CF can be targeted (Bell et al., 2020; Zemanick and Accurso, 2019). At the time of this research, four CFTR modulators were approved, with the most recent being a triple combination of three different compounds, Elexacaftor, Tezacaftor and Ivacaftor (ETI) also known by its brand names Kaftrio or Trikafta. Since then, a new triple combination therapy, Vanzacaftor/Tezacaftor/Deutivacaftor, has been approved (Southern, 2025). This study focuses on Elexacaftor/Tezacaftor/Ivacaftor which will be referred to by its generic term ‘ETI’. ETI has been authorised for use in healthcare systems in many medium high-income countries such as the UK in 2020 (Bierlaagh et al., 2021; Hine et al., 2022). ETI targets the most common CF mutation, potentially benefitting approximately 90% of people within the CF population (Hine et al., 2022). Whilst there is growing research on the physical and psychological benefits of ETI, little is known about the experiences of people with CF who cannot benefit from ETI due to genetic factors. The current study focuses on the experiences of this group.

### Experiences of those suitable for ETI: The 90%

Since the release of ETI, there has been an emergence of qualitative research exploring the experiences of people with CF benefitting from this modulator therapy. Keyte et al. (2023), Aspinall et al. (2022), Almulhem et al. (2022) and Page et al. (2022) collectively reported positive outcomes such as improved physical wellbeing, quality of life, lung function, weight gain and an increased sense of control, optimism, hope for the future in some participants, newfound abilities to engage in activities and hopes to reach new milestones, like parenthood and retirement. Participants in these studies also reported the negative impact of CFTR modulators, including adverse mental and physical side effects, feelings of loss or the need to redefine one’s identity, heightened anxiety and distress following health changes, a perceived lack of understanding from their clinical teams and feelings of survivors’ guilt about benefitting from ETI (Aspinall et al., 2022; Dagenais et al., 2020; Keyte et al., 2023; Talwalkar et al., 2017).

### Experiences of those not suitable for ETI: The 10%

Various factors contribute to why certain people with CF cannot benefit from ETI. These include having rare mutations, intolerance to the drug, limited access due to costs, age-related restrictions (ETI is presently approved for individuals aged 2 years and above in the UK, but is likely to be different in other countries; Cystic Fibrosis Trust, 2023) and exclusion post-lung transplant as modulators are not routinely recommended for this population (Cystic Fibrosis Trust, 2022; Desai et al., 2022; Fajac and Sermet, 2021).

Only two studies have investigated the experiences of people with CF who are not able to benefit from ETI. In a survey distributed through social media and email, Kramer-Golinkoff et al. (2022) found that although most were happy for those benefitting from ETI, they expressed a strong desire to experience similar improvements, resulting in feelings of being overlooked alongside negative self-comparisons with those benefitting from ETI. Secondly, Milo et al. (2023) reported that Italian participants in a qualitative study using thematic analysis expressed conflicting emotions about their declining health and feelings of happiness when observing others’ successes. They described a sudden shift from hope and enthusiasm prior to the release of ETI, to disappointment when they found out that the type of mutation they had did not meet the criteria for ETI. They also felt a lack of hope for the future while simultaneously holding onto a renewed sense of hope in relation to ongoing medical research. However, since this study focused on a younger Italian demographic, exploring perspectives of older individuals with CF beyond this group could offer new insights. Additionally, understanding how people with CF who are not able to benefit from ETI re-interpret and find meaning in their situation, remains an important area for further investigation.

This study focused on adults with CF who cannot benefit from ETI due to genetic factors and seeks to understand how these individuals’ make sense of this situation. This understanding has significant research and clinical practice implications.

## Methods

### Research design

Interpretative Phenomenological Analysis (IPA) was chosen as the methodology for this study to better understand individual participants’ experiences by exploring how they make sense of significant life experiences (Smith et al., 2021). The theoretical framework for IPA integrates phenomenology, which explores participants’ lived experience; hermeneutics, which focuses on interpretation; and idiography, which looks closely at each participants’ experience in depth before looking across cases and identifying broader patterns (Smith et al., 2009, 2021). A key feature of IPA is the hermeneutic circle, which involves moving between parts and the whole. It also includes a double hermeneutic process, where participants make sense of their experiences and researchers interpret this meaning-making (Smith et al., 2021). Given this, the role of the researcher is important. The researcher aims to understand the participant’s experience as directly as possible, setting aside personal biases (Smith et al., 2021).

### Ethical considerations

Ethics approval was gained from Cardiff University School of Research Ethics Committee on 7th December 2023 and from one UK NHS Health Board Research and Development Department on 10th October 2023. Participants gave written informed consent and signature before starting interviews.

### Participants

Participants were eligible if they were: over 18; unable to benefit from ETI due to genetic factors; sufficiently fluent in English to take part, and they self-confirmed that they were well enough physically and mentally to participate in an interview. Participants who were acutely unwell or experiencing an acute exacerbation were excluded. Seven adult CF participants – four males and three females – took part in the study. Participants were aged 19–61; their mean lung function was 65.57% (predicted FEV1), and some participants were from racially diverse backgrounds. Participants were assigned gender neutral pseudonyms and referred to with gender-neutral pronouns. Demographic details have been aggregated to protect participants from identification.

Participant recruitment took place through two streams. For the first recruitment stream, participants were recruited internationally through CF charities, online CF support groups and various social media platforms using poster advertisements. For the second recruitment stream, the Principal Investigator (PI), who was a Clinical Psychologist, along with Research Specialist Nurses within a UK NHS CF Service identified potentially suitable patients from their clinical databases. Recruitment was conducted from March 2023 to May 2024. Four participants were recruited through recruitment stream 1 and 3 through stream 2, six were UK based and one from Australia. Information sheets were provided, and written informed consent was obtained from all participants to take part in the study and publication of its findings.

Semi structured interviews were conducted by the first author online via Teams, lasting between 35 and 70 minutes. The potentially sensitive nature of the research topic was acknowledged, and participants were advised that they did not have to answer any questions they did not wish to. They were also told that they could take a break during the interview if necessary. Socio demographic information such as age, gender, lung function, ethnicity and location were also gathered to situate the sample. Table 1 shows the core interview questions.

**Table 1.** Core interview questions (additional semi-structured prompts were given to allow participants to elaborate).

Interview questions
1. Can you tell me about your experience of not being able to benefit from ETI? 2. Are you able to say a bit about what it feels like to not be suitable for receiving ETI? 3. What has life been like for you since you found out? 4. What does being unable to benefit from ETI mean for you? 5. Has this made a difference on how you view your life or things that are important to you? 6. How do you see yourself in the future? 7. Is there anything else that you feel is important that you have not yet had the opportunity to say?

The schedule was developed in consultation with the existing literature, discussions among the research team and staff and service users at the CF Trust. The schedule served as a guide for participants to prioritise and share what they considered to be central to their experience of not benefitting from ETI. The style of the interview was inductive, fostering reflection and exploration and prompts were used where necessary.

At the end of the interview, a debrief session was given and although none of the participants expressed distress during the interview, they were signposted to relevant sources of support if needed.

### Data analysis procedure

All interviews were audio recorded via Teams with participants’ permission. Six interviews were transcribed verbatim by the first author and one recording was submitted to a professional transcription service, due to time constraints and a confidentiality agreement was signed. The first author read the transcripts multiple times or re-listened to the audio recordings and made exploratory notes, commenting on the participants’ descriptions, language and conceptual elements, particularly focussing on overall concepts or psychological aspects (Smith et al., 2021). Audio recordings were deleted within 1 week of a transcript being produced.

Following these exploratory notes, the first author constructed experiential statements that highlighted the key features from the transcript and then identified connections across experiential statements within each transcript, grouping some statements together and noting overarching concepts. Statements were cross referenced in the transcript to ensure they were grounded in the participants’ specific words and cluster of experiential statements was termed as the participants ‘Personal Experiential Theme’ (Smith et al., 2021).

 Once all the seven transcripts were analysed, the first author then looked for patterns of similarity and differences across participants’ ‘Personal Experiential Themes’ generating a set of ‘Group Experiential Themes’ (Smith et al., 2021). The analysis continued into the writing up process, revealing patterns of convergence and divergence (Smith et al., 2021).

### Reflexivity

The first author kept a reflexive diary throughout the research process, documenting thoughts, reflections and emerging connections. These were regularly explored in research supervision during their doctorate training in Clinical Psychology. After engaging with interviews, the first author was moved by the experiences shared by participants, and their gratitude for the research. These emotional responses were discussed to ensure they informed, but did not unduly shape the analysis.

## Findings

From the analysis of seven transcripts, four themes emerged. Words that have been omitted from quotes are indicated by ellipses ‘. . .’ and words added to enhance clarity and understanding for the reader are in square brackets ‘[]’. What follows is a detailed description of each ‘Group Experiential Theme’, supported by quotes from participants. Participants referred to ETI by its brand names Kaftrio and Trikafta.

### Theme 1: Feeling forgotten

A significant theme highlighted in participants’ descriptions was ‘feeling forgotten’. With the release of ETI gaining a significant amount of media coverage, some participants stated:

. . .and all the stuff in the media is focused on Trikafta Trikafta, and we sort of get a bit forgotten about all those who can’t benefit. . . (Bailey)

. . .we are sort of left out of discussion when we talk about, you know, a cure for CF or a better medication for it [CF]. . . (Sam)

Jamie highlighted discussions about those who cannot benefit from ETI as an afterthought, which further emphasises feelings of being overlooked. This was linked to an increase in feelings of frustration, possibly due to the expectation that research presenters should have a better understanding of inclusivity in their practice.

. . .like a final note at the end of a presentation is, of course we know that this doesn’t apply to everyone, and there are a few people that don’t benefit from this, its things like that that just add to the frustration sometimes . . . (Jamie)

Jamie further highlighted gaps in services such as ‘theratyping’, which matches medications to specific types of mutations (Clancy et al., 2019). The absence of established systems and support added to the feeling of being forgotten, overlooked, frustrated and undervalued.

. . .It’s [lack of services and information] just another thing that you don’t have access to. It’s just, it’s an annoyance, it’s a frustration. . . (Jamie)

Two participants did not express feeling forgotten, perhaps, due to reportedly being in a better health position than the other five participants. They stated:

To be honest, my – my health tends to be quite – I am quite healthy with CF, you know, all things considered. (Frankie)

. . . I’m not suffering with Cystic Fibrosis on a chronic basis. . .I’m lucky. . . (Ashley)

#### ‘Us and them’ comparisons

Visibility on social media and TV shows prompted four participants to compare themselves with those who can benefit from ETI, leading to feelings of being forgotten and a sense of missing out which seemed to enhance their struggle.

. . .when the ‘this changed my life’ stories came into social media at the same time that I started being ill, I just felt like it’s something I didn’t want to hear about. So I blocked certain keywords on all of my profiles. Anything to do with [CF] or Kaftrio. . . (Jamie)

Social media served as a reminder that other people’s lives were improving whilst theirs was not. For Jamie, witnessing the improvement in others’ lives while theirs was deteriorating seemed to engender feelings of sadness, loss and an overwhelming sense that they could only cope by avoiding CF or ETI related information. There was also a sense of disbelief at the improvements. For Bailey, there was a significant contrast between their life and the life of the individuals benefitting from ETI that they saw on a TV show:

. . .Oh my gosh! Like, is she kidding? She doesn’t have to do this anymore. . .Oh why me? Like the amount of hours I spend a day doing a nebuliser. Imagine just not having to do that anymore and just taking 3 pills in the morning or whatever and just going on with my day like a normal human. That just seems sort of incomprehensible to me. . . (Bailey)

Here, the participant on the show appears to experience a reduced treatment burden, a sense of normality and freedom from CF-related constraints. Although Bailey did not explicitly mention feeling resentful, there is a sense that they might have these feelings as they compare their need for three nebulisers to what they perceive as the ease of other treatments. Bailey’s questioning of ‘why me’ implies a sense of unfairness and injustice, feeling that others benefit from something they cannot. Three participants expressed indifference towards ETI. They conveyed that ETI held no significance for them, reflecting acceptance and a focus on moving forward.

#### Lack of consideration in research priorities

Four participants also highlighted feeling forgotten in discussions about research priorities. Participants highlighted issues around funding and feeling like research efforts would not be directed to a minority group:

. . .who on Earth is waking up one day going ‘Oh, let me decide to research it [a drug that can benefit people with CF not able to benefit from ETI]’? I mean, why wouldn’t someone want to join the team that’s researching Trikafta that they probably make a lot more money. (Bailey)

These feelings of being overlooked in research priorities due to funding were also connected to feelings of not being seen as worthy or seeing themselves as a poor investment. This highlights a sense of being excluded or undervalued, as if they are not considered worthy or enough to justify funding. There was an underlying sense that these emotions may have been present, given the perceived injustice and unfairness of the situation.

#### Variable effort from healthcare professionals (HCPs)

All participants highlighted having positive and supportive relationships with their CF teams. However, not all interactions with HCPs were positive, for example:

. . .I’ll do anything that helps you know people like me, but . . . from the other side, from the NHS, from the medical professionals [there’s] just not seeing the same [effort] that’s just heartbreaking you know. . . (Sam)

Participants in the current study conveyed a sense of being forgotten, frustration and disappointment due to what they perceived as insufficient information, insensitivity and a lack of commitment from HCPs. Describing the experience as ‘heartbreaking’ suggests a strong emotional reaction to feeling that their efforts are not met with the same level of reciprocity.

### Theme 2: Conflicting emotions

This theme focusses on the conflicting emotions that arose for participants when reflecting on their situation and others’ who can benefit from ETI.

. . .I’m very happy for those who can [benefit from ETI]. . . but then it also creates this level of disappointment that, yes, it’s been great for them, but it’s not something that I can benefit from. . . (Sam)

Disappointment stems from realising that ETI is something they cannot benefit from, highlighting a longing and desire for something they cannot have. Jamie echoed this sentiment:

. . .I was as happy for them as anyone could be, but it’s then when the ‘this changed my life’ stories came into social media at the same time that I started being ill, I just felt like it’s something I didn’t want to hear about. . . (Jamie)

These feelings of jealousy, along with emotions like excitement and happiness, were also shared by Bailey:

. . . when I found out it wouldn’t work, it was just sort of. . . felt like another setback in a way. . .but obviously it’s mixed with feelings of incredible excitement for those who could benefit. But I obviously, selfishly wanted to be able to benefit as well. . . (Bailey)

The use of the word ‘another setback’ illustrate the multiple challenges that come with CF, with not benefitting from ETI being an additional difficulty. In contrast, Alex expressed happiness and did indicate sadness about not having something similar to benefit from.

. . .I’m just kinda glad that there is something for the younger people, you know, or anybody. . . I’m not bitter. I’m not angry, I’m a little sad that there isn’t something that I could have a go at, but it’s no more than that. . . (Alex)

Unlike the experiences shared by other participants, two participants primarily conveyed positive emotions regarding the benefits of ETI for others. They stated:

. . .And for me, it’s absolutely brilliant news. And I’m very buoyant about that. And I’m very happy. . . (Ashley)

. . .I’ve got friends who have a child with CF, who has the Kaftrio drug, and they’ve said it’s life-changing for them, which is great at the end of the day. (Frankie)

The more positive responses of these participants may be linked to their relatively better health status. However, Frankie and Alex did acknowledge that they may have felt differently if their situation was different.

You know, it’s – I think it’s – if I was ill all the time, I think I’d probably have a different outlook on it [ETI] and I think it would affect me a lot more. (Frankie)

 . . .as things [health] get worse. . .I will maybe feel a little bit more angry or anxious. . . (Alex)

This theme highlights the nuances present even within a small subgroup like the 10% who cannot benefit from ETI. The conflicting emotions they experience seemed to be influenced by their individual health situations.

### Theme 3: Fragility of hope

Hopeful experiences were intertwined throughout and reported by all seven participants. While five participants initially harboured hope upon ETI’s release, believing they could benefit, discovering their rare mutations took the hope away.

. . .it was a bit disappointing. . .I’ve always been told that. . .there’s always that gene therapy going on and one day it’s going to be available. . . I’ve always had that hope in the back of my mind. . .then to be sort of told that I’m not eligible for it [ETI]. . . (Charlie)

Bailey further discussed a recurring pattern and the familiarity of things not working out, followed by the disappointment of not being able to benefit from something that benefits most of the CF population.

. . .But I feel like nothing ever really seems to happen or to work. And then finally, this thing did work. But then of course, there’s a catch. And it didn’t work for me. And I just sort of felt like, oh, this is so typical. (Bailey)

It seems that Bailey began with little hope or scepticism about a successful drug but the surprise that ETI did work initially brought a sense of hope, followed by disappointment and the loss of hope that it would not work for them.

Other participants expressed concerns about their current health status, which might not allow them to benefit from new drugs.

And I just keep hoping that maybe they will find something that I can have a go at before it gets to the point where maybe I wouldn’t get any benefit. (Alex)

Three participants emphasised the importance of maintaining hope but expressed concerns about declining health or possibility of death.

#### Hope enduring

Although participants mentioned experiencing a loss of hope, they also shared instances where they still had hope. All participants recognised the value of this research and were grateful for the opportunity to talk about their experiences. Additionally, two participants mentioned how this research had instilled hope.

. . .and then I stumbled upon this study for CF. And I thought, you know, this shines a light on people like me who have been, you know, left out of the discussion. . . (Sam)

This research appears to have not only made this participant feel acknowledged and provided an opportunity for their voice to be heard, but also instilled a sense of hope that they were not overlooked. This feeling was also expressed by another participant who, upon questioning if anyone would research rare mutations, shared:

. . .but then I mean, you’ve given me hope because I thought who on Earth would want to know about the psychological impacts of someone that can’t benefit from Trikafta. So, you know, thank you again for doing this. (Bailey)

These extracts demonstrated how research has the power to instil hope in marginalised groups who may find themselves in situations of hopelessness. Additionally, all seven participants expressed a shared belief that the introduction of ETI had sparked hope for upcoming treatments:

. . .I thought, you know, the fact that we have this [ETI], I was optimistic to some extent that we have been able to research this, this miracle of a drug and going forward, we might even come up with something that does help people with rare mutations. . . (Sam)

Sam acknowledged the CF community’s prolonged anticipation for a drug like ETI, noting that the wait had been extended for them due to their inability to benefit from ETI. Despite this, they remain optimistic and hopeful for the future, expressing a strong belief in the development of a new drug for individuals with rare mutations. Another participant expressed similar feelings of hope while also voicing concerns about what it means in terms of research priorities:

. . .Yeah, I think a little bit of both, but I think definitely Trikafta has given hope. . . but at the same time, it’s also, in a sense, made me concerned that all the research will now be focused on that mutation. But, I still do hold out hope because you know, if it can happen for some people, why not me? (Bailey)

### Theme 4: Remaining on the old CF trajectory

A notable aspect of the experiences of individuals with CF who cannot benefit from ETI was the feeling that they remained on the old CF trajectory. Bailey mentioned that those taking ETI no longer had to be concerned about their health:

. . .yeah, I think since Trikafta it’s definitely made it more apparent that I’m still on the old track of CF disease progression where people get worse, not better. (Bailey)

This extract recognises the positive trajectory for individuals who can benefit from ETI, highlighting the contrast that made it clearer that Bailey was still experiencing the old course of CF disease progression, with the certainty of health decline.

Another participant draws a sharp comparison between the lives of individuals with rare mutations and those who cannot benefit from ETI:

. . .It is literally a life changing thing for those who can benefit from it. . . but for those who can’t benefit from it, they have to, you know, continue with all their treatments and still have a not-so-great quality of life. . . (Sam)

Again, this extract emphasises feelings of being left behind, and highlights the impact of not benefitting from ETI, resulting in diminished quality of life. Being in this situation also has the potential to influence one’s sense of identity regarding deserving treatments or not.

Two participants reflected on their health declining whilst seeing others’ health improving, and a sense of ‘cruel timing’ seeing others improve whilst they remain on the old CF trajectory:

. . .like it’s really cruel timing in the way it’s worked out that the majority of people with CF are now having a better life and CF isn’t affecting them as much as it was. At the same time the exact opposite happened to me. . . (Jamie)

. . .because no matter how much I fight this illness. How much I get out of bed and do my physio and clear my lungs. . .I’m not really feeling the benefit of it anymore. . . (Charlie)

Here, Charlie suggests that their efforts are insufficient, leading to feelings of helplessness and an urgent desire for something beneficial as the current therapies do not appear to provide the needed help. Another participant recognised the urgency stemming from declining health and the absence of current options for improvement:

. . .but my CF, my lung damage is quite severe now, it’s progressing, so I’m not sure again how much if I did get the option it would actually change that now. (Alex)

This extract emphasises the certainty of health declining and the uncertainty of a new drug that could help with this decline of health, a feeling echoed in other participants’ narratives.

Two participants expressed feelings of unfairness and injustice due to their experience of following the old trajectory of CF. This shifted their perspective from seeing themselves as part of the group unable to benefit from ETI to simply being individuals living with CF. Bailey expressed feelings of unfairness using a metaphor involving a deck of cards:

. . . I feel like in general just being dealt like the cystic fibrosis hand (laughs) if it was a deck of cards, I didn’t get [. . .] dealt the best deck. Or the best hand. . . (Bailey)

This metaphor may suggest the unfairness of life and the added difficulties that accompany not benefitting from ETI, on top of the challenges of having CF. Jamie also echoed this feeling employing a metaphor involving bingo:

. . .If I played CF bingo, I’d win, so I’m having [list of health difficulties]. It’s like everything’s come all at once. In the modulator drugs . . . you take them and they circulated through your body. . . so it benefits all of the areas of CF in your body. It’s not just your lungs. . . (Jamie)

Whilst all the participants acknowledged to an extent that they were on the old path of CF disease progression, the distinguishing factor now is that they can compare their situation with others who are receiving benefits from ETI, making their situation worse.

#### Distraction and positivity as a coping mechanism

Various coping strategies were integrated into the narratives of different participants. Distraction and maintaining positivity emerged as common coping mechanism for some participants. Jamie, Alex and Frankie highlighted that:

. . .I try not to think about the fact that it’s not working, that I don’t have that benefit that other people have and try and distract myself by looking at things that I can do to potentially change that. . . (Jamie)

. . .I just sort of shake myself and just sort of think. . .there’s always people that I’m close to that unfortunately. . . that are struggling more and I think just put it into context where you are, you know and if you’re not well. . .do something proactive. (Alex)

In this instance, these participants seem to engage in perspective taking; there is a sense of empathy and compassion towards others, and they advocate taking proactive steps to address one’s health and wellbeing. There’s a notable sense of empowerment and agency in taking control of their life. There is a feeling of embracing the current situation and discovering happiness and satisfaction in the present moment. Despite facing health challenges, there is a sense of appreciation and fulfilment, along with the deliberate decision to enjoy their day-to-day life. All participants expressed a sense of positivity as a coping strategy.

. . . I’m generally a positive person. I don’t dwell on like negativity for too long. . .especially with CF, you can’t you’ll have good days, and you’ll have bad days. Mostly you’ll have bad days. (Bailey)

In terms of Kaftrio, I’ve moved on from it totally because I know it’s something that I can’t take. You know, there’s no point, once again, there’s no point dwelling on it. (Frankie)

There’s an awareness of negative events occurring, yet there’s also a tendency to avoid dwelling on them. Other participants also emphasised the importance of maintaining a sense of positivity or optimism as a coping strategy.

. . .I’m very blessed and I always try and stay optimistic. And I know I’m a lot healthier than, you know, a lot of people in the world. (Bailey)

Finding solace in comparing their health to others and perceiving themselves as ‘healthier’ seems to contribute to their coping mechanism as well.

I always just seem to have quite a positive outlook on life in general. (Frankie)

. . .You have to live life to your fullest, you know? And that’s what I try to do most the time. But it’s just that you always have this thing in the back of your mind that . . . you’d always . . . have to struggle. . . (Sam)

There is a notable sense of positivity and resilience in persisting despite challenges of CF and not experiencing benefits from ETI. However, it appears that for Sam, it is not always possible to maintain this mindset successfully, as the thought of not benefitting from ETI often lingers in their mind.

## Discussion

This study explores how adults with CF who are not able to benefit from ETI due to genetic factors make sense of their experiences. This is the first study to use an IPA methodology to explore these experiences. The study identified four main themes, including ‘feeling forgotten’, ‘conflicting emotions’, ‘the fragility of hope’ and ‘remaining on the old CF trajectory’. This group has often been referred to as the ‘10%’ (Desai et al., 2022), however, the current study offered a much-needed nuanced perspective of the experiences that exist within this group.

While all participants shared in the inability to benefit from ETI due to genetic factors, their individual situations varied. Some were relatively healthier, some learned upon ETI’s release that their mutations had been misidentified, placing them in the ‘10%’, some had late CF diagnoses and were grateful for the ‘good years’ they had, while others experienced health deterioration post ETI release. Despite feeling forgotten, grappling with a range of emotions, and recognising that they had remained on the old path of CF disease progression, all participants acknowledged the transformative potential of ETI and held on to hope for future treatments.

The first theme, ‘feeling forgotten’ emerged as a prominent experience among participants, aligning with findings from Kramer-Golinkoff et al. (2022), who conducted a survey with people with CF and their families, about their experiences of not being able to benefit from ETI. While their survey included open-ended questions, it was limited in its ability to capture the depth of participants’ experiences. Additionally, the Kramer-Golinkoff et al. (2022) study combined the experiences of individuals with CF and their caregiver’s, potentially limiting the specific experiences of those with CF who cannot benefit from ETI.

This study extends previous research by capturing nuanced emotions interlinked with feeling forgotten, such as disappointment and frustration. The current study extends findings reported by Milo et al. (2023), as the use of IPA provided the opportunity for an in-depth exploration of participants experiences. Some participants in the current study felt overlooked in research priorities due to funding issues, leading to a perception of being seen as a poor investment. This sense of unworthiness, fuelled by a lack of research funding, echoes concerns raised by Landess et al. (2024) in a qualitative study primarily involving individuals who can take CFTR modulators, with one participant who couldn’t benefit from ETI reported fear that less prevalent mutations would attract less research interest.

One participant in the current study felt that being in the minority group without access to drugs like ETI made their challenges more pronounced compared to those benefitting from ETI, who, despite ongoing challenges, experience a better quality of life. This highlighted a profound sense of being left behind and highlights the need for increased funding and advocacy for this group. Their experiences also raise important questions about the value placed on their lives and the persistent feelings of being forgotten and unworthy in health economic decisions (Brendbekken and Bhopal, 2024; Wise, 2023).

Despite feeling forgotten, participants expressed a strong desire to be involved in research and trials for treatments, aligning with Kramer-Golinkoff et al. (2022). However, this study uncovered deeper themes not captured by previous research, revealing varied experiences regarding information about ETI and new treatments. Some participants in the current study felt that HCPs did not provide adequate information about ETI upon its release and lacked commitment to updating them on new trials.

 The second theme of this study ‘conflicted emotions’ revealed a complex mixture of emotions among people with CF who cannot benefit from ETI. Participants in the current study expressed happiness and excitement for those who could benefit, alongside disappointment, sadness, feeling overwhelmed, and a strong desire to experience similar benefits themselves. These emotions were intertwined with a sense of hope. This contrasts with studies on those who can benefit from ETI, who often feel happy for themselves but sad for those who cannot (Aspinall et al., 2022; Keyte et al., 2023; Page et al., 2022). These conflicting emotions reflect the close-knit nature of the CF community while also revealing potential divisions because of ETI. One participant in the current study noted that media focus on ETI risked overshadowing the needs of those who cannot benefit, while another reported avoiding any stimuli.

The third theme, ‘the fragility of hope’, captured the fluctuating sense of hope experienced by people with CF who cannot benefit from ETI due to genetic factors. While hope was a constant in their lives, participants initially felt hopeful about ETI’s release, only to quickly lose that hope upon discovering their genetic mutations excluded them from its benefits. This led to feelings of disappointment and disbelief, with one participant questioning ‘why me?’ This mixed sentiment aligns with findings reported by Milo et al. (2023). The use of IPA methodology in this study was particularly effective in capturing the nuanced fluctuations of hope, and the detailed, personal transitions in participants’ experiences.

The final theme revealed that participants felt that they had remained on the old CF trajectory, facing worsening health typical of CF. They recognised the significant impact ETI could have on their overall wellbeing if they were eligible to receive it. Participants also expressed a sense of unfairness and injustice regarding the continued burden of their current treatments with limited efficacy. Participants in the current study employed coping strategies such as distraction, avoidance, optimism and maintaining a positive attitude. White et al. (2018) reviewed existing literature on coping with chronic illness and proposed a framework for clinicians. The framework included internal factors such as personal habits, individual differences and preferences, values and belief and emotional factors, as well as external resources such as social support and therapeutic interventions. White et al. (2018) emphasised the importance of both internal and external factors in promoting positive adjustment and coping. For instance, social support from family, friends and interactions with others who cannot benefit from ETI can help alleviate feelings of isolation, being left behind, or unworthiness reported in the current study. Moreover, psychological interventions could assist people with CF who are not able to benefit from ETI by offering strategies to manage, regulate and express emotions, as suppressing or avoiding emotional responses is linked to poor coping outcomes (de Ridder et al., 2008).

### Study strengths and limitations

The study methodology, IPA, enabled a nuanced account of the experiences of a small and under-researched target group to be examined in depth. Through detailed interviews and iterative interpretative analysis, the study critically interpreted and explored underlying meanings. While previous studies have examined the experiences of this group, they lacked this level of analysis and interpretation, marking a unique contribution to the existing literature. Information was gathered through semi structured interviews, allowing the researcher to follow different lines of inquiry as they naturally arose. The seven participants represented various geographical locations within the UK and Australia, providing a broader perspective than studies that focused on a single locality, such as Milo et al. (2023), which centred on an Italian demographic.

The current study faced limitations, primarily the small sample size of seven participants. Despite challenges in recruiting, the sample size met the minimum criteria for an IPA study. Another limitation of the study is that most of the participants were from the UK, with one from Australia and although generalisability is not the aim of an IPA study, this potentially limits the applicability of findings beyond these regions, particularly to those in medium-low-income countries. Additionally, the study did not include views from those unable to benefit from ETI due to other factors like lung transplant or limited access in their countries.

### Clinical and research implications

Participants in this study generally reported positive relationships with their CF healthcare teams, but also expressed disappointment over gaps in service provision and information about ETI, new clinical trials and new research. They emphasised that better communication could help them feel more acknowledged and included. One participant in the current study, for instance, did not feel like part of the ‘forgotten few’ due to their active participation and awareness of ongoing developments for people who cannot benefit from ETI. HCPs should consider acknowledging the specific struggles faced by people with CF not able to benefit from ETI, ensuring that they do not feel ‘forgotten’. They should communicate the reasons for ETI ineligibility clearly and empathetically, acknowledging the patient’s disappointment and other emotions. Clinicians should also provide updates on ongoing research and potential future therapies to help mitigate feelings of hopelessness. Service users unable to benefit from ETI could offer peer support, fostering community, validation and reducing feelings of being forgotten and unworthy.

Given the different emotions experienced by participants in this study, such as hopelessness, feeling forgotten, undervalued and powerless, it seems important that people with CF not suitable for ETI are offered psychological support where possible. In the wider CF literature, psychological support has been shown to be helpful (Havermans and Duff, 2020; Havermans and Staab, 2016). Interventions such as Acceptance and Commitment Therapy (ACT) and mindfulness exercises could be helpful for managing difficult and conflicting emotions associated with not being able to benefit from ETI, and might lead to a greater sense of living in the present moment and acceptance (Havermans and Duff, 2020; Kauser et al., 2022).

Participants in the current study raised concerns about media portrayals of CF and ETI potentially leading to misconceptions and diverting funding away from research that could benefit them. They called for fair and equitable access to effective treatments for all people with CF. This suggests that there could be a role for CF charities to work with the media to ensure balanced portrayals that include the experiences of those not eligible for ETI and could involve featuring diverse patient stories and advocating for inclusive research and funding approaches. Additionally, engaging service users in setting research priorities and advocacy efforts could ensure that the most pressing unmet needs are addressed.

The approval of Vanzacaftor/Tezacaftor/Deutivacaftor has marked the next generation of CFTR modulator therapies (Southern, 2025). While the new drug offers hope for some people with CF who previously were unable to benefit from ETI, a minority will remain without effective options, hence there will be a continued need for research and support for this group.

## Conclusion

This research explored the experiences of adults with CF who cannot benefit from ETI due to genetic factors. Participants in the current study shared rich, emotive, and personal stories that hold significant value for HCPs, therapists, researchers, pharmaceutical companies and government organisations. Participants accounts highlight the profound impact of not benefitting from ETI, particularly feeling forgotten and undervalued, yet holding on to hope. Understanding these perspectives can guide the development of more inclusive and effective support systems and treatments.

## References

[bibr1-13591053251369393] Almulhem M Harnett N Graham S , et al. (2022) Exploring the impact of elexacaftor-tezacaftor-ivacaftor treatment on opinions regarding airway clearance techniques and nebulisers: TEMPO, a qualitative study in children with cystic fibrosis, their families and healthcare professionals. BMJ Open Respiratory Research 9(1): e001420. 10.1136/bmjresp-2022-001420 PMC9557266 36207030

[bibr2-13591053251369393] Aspinall SA Mackintosh KA Hill DM , et al. (2022) Evaluating the effect of Kaftrio on perspectives of health and wellbeing in individuals with cystic fibrosis. International Journal of Environmental Research and Public Health 19(10): 6114. 35627651 10.3390/ijerph19106114 PMC9141876

[bibr3-13591053251369393] Bell SC Mall MA Gutierrez H , et al. (2020) The future of cystic fibrosis care: A global perspective. Lancet Respiratory Medicine 8(1): 65–124. 31570318 10.1016/S2213-2600(19)30337-6 PMC8862661

[bibr4-13591053251369393] Bierlaagh MC Muilwijk D Beekman JM , et al. (2021) A new era for people with cystic fibrosis. European Journal of Pediatrics 180(9): 2731–2739. 34213646 10.1007/s00431-021-04168-y PMC8346380

[bibr5-13591053251369393] Brendbekken A Bhopal A (2024) Putting Kaftrio into perspective: A test case for fair and open priority setting. BMJ 384: 384. 10.1136/bmj.q182 38286477

[bibr6-13591053251369393] Chen Q Shen Y Zheng J (2021) A review of cystic fibrosis: Basic and clinical aspects. Animal Models and Experimental Medicine 4(3): 220–232. 34557648 10.1002/ame2.12180 PMC8446696

[bibr7-13591053251369393] Clancy JP Cotton CU Donaldson SH , et al. (2019) CFTR modulator theratyping: Current status, gaps and future directions. Journal of Cystic Fibrosis 18(1): 22–34. 29934203 10.1016/j.jcf.2018.05.004 PMC6301143

[bibr8-13591053251369393] Cystic Fibrosis Trust (2022) Kaftrio – complex and individual experiences. Available at: https://www.cysticfibrosis.org.uk/sites/default/files/2022-04/Kaftrio%20-%20complex%20and%20invidual%20experiences.pdf (accessed 26 June 2025).

[bibr9-13591053251369393] Cystic Fibrosis Trust (2023) Kaftrio now approved for 2–5-year-olds. Available at: https://www.cysticfibrosis.org.uk/news/kaftrio-now-approved-for-2-5-year-olds (accessed 26 June 2025).

[bibr10-13591053251369393] Dagenais RVE Su VCH Quon BS (2020) Real-world safety of CFTR modulators in the treatment of cystic fibrosis: A systematic review. Journal of Clinical Medicine 10(1): 23. 33374882 10.3390/jcm10010023 PMC7795777

[bibr11-13591053251369393] Davies G Rowbotham NJ Smith S , et al. (2020) Characterising burden of treatment in cystic fibrosis to identify priority areas for clinical trials. Journal of Cystic Fibrosis 19(3): 499–502. 31735561 10.1016/j.jcf.2019.10.025

[bibr12-13591053251369393] de Ridder D Geenen R Kuijer R , et al. (2008) Psychological adjustment to chronic disease. Lancet 372(9634): 246–255. 18640461 10.1016/S0140-6736(08)61078-8

[bibr13-13591053251369393] Desai M Hine C Whitehouse JL , et al. (2022) Who are the 10%? - Non eligibility of cystic fibrosis (CF) patients for highly effective modulator therapies. Respiratory Medicine 199: 106878. 35633605 10.1016/j.rmed.2022.106878

[bibr14-13591053251369393] Fajac I Sermet I (2021) Therapeutic approaches for patients with cystic fibrosis not eligible for current CFTR modulators. Cells 10(10): 2793. 34685773 10.3390/cells10102793 PMC8534516

[bibr15-13591053251369393] Goldbeck L Fidika A Herle M , et al. (2014) Psychological interventions for individuals with cystic fibrosis and their families. Cochrane Database of Systematic Reviews 2014(6): CD003148. 10.1002/14651858.CD003148.pub3 PMC7388585 24941199

[bibr16-13591053251369393] Havermans T Duff AJA (2020) Changing landscape: Psychological care in the era of cystic fibrosis transmembrane conductance regulator modulators. Current Opinion in Pulmonary Medicine 26(6): 696–701. 32941351 10.1097/MCP.0000000000000727

[bibr17-13591053251369393] Havermans T Staab D (2016) Mental health in cystic fibrosis: turning the tide. Thorax 71(1): 1–2. 26678432 10.1136/thoraxjnl-2015-208076

[bibr18-13591053251369393] Hine C Nagakumar P Desai M (2022) Small molecule drugs in cystic fibrosis. Archives of Disease in Childhood – Education and Practice 107(5): 379–382. 10.1136/archdischild-2020-319009 33214241

[bibr19-13591053251369393] Hisert KB Birket SE Clancy JP , et al. (2023) Understanding and addressing the needs of people with cystic fibrosis in the era of CFTR modulator therapy. Lancet Respiratory Medicine 11(10): 916–931. 37699420 10.1016/S2213-2600(23)00324-7 PMC13005650

[bibr20-13591053251369393] Kauser S Keyte R Mantzios M , et al. (2022) A qualitative exploration into experiences and attitudes regarding psychosocial challenges, self-compassion, and mindfulness in a population of adults with cystic fibrosis. Journal of Clinical Psychology in Medical Settings 29(4): 898–910. 35147829 10.1007/s10880-022-09859-8 PMC9636098

[bibr21-13591053251369393] Keyte R Kauser S Mantzios M , et al. (2023) The psychological implications and health risks of cystic fibrosis pre- and post-CFTR modulator therapy. Chronic Illness 19(3): 539–556. 35502821 10.1177/17423953221099042

[bibr22-13591053251369393] Kramer-Golinkoff E Camacho A Kramer L , et al. (2022) A survey: understanding the health and perspectives of people with CF not benefiting from CFTR modulators. Pediatric Pulmonology 57(5): 1253–1261. 35170259 10.1002/ppul.25859 PMC9314897

[bibr23-13591053251369393] Landess L Prieur MG Brown AR , et al. (2024) Exploring perceptions of and decision-making about CFTR modulators. Pediatric Pulmonology 59(6): 1614–1621. 38456611 10.1002/ppul.26953

[bibr24-13591053251369393] Milo F Ciciriello F Alghisi F , et al. (2023) Lived experiences of people with cystic fibrosis that were not eligible for elexacaftor-tezacaftor-ivacaftor (ETI): A qualitative study. Journal of Cystic Fibrosis 22(3): 414–419. 36549989 10.1016/j.jcf.2022.12.007

[bibr25-13591053251369393] Page A Goldenberg A Matthews AL (2022) Lived experiences of individuals with cystic fibrosis on CFTR-modulators. BMC Pulmonary Medicine 22(1): 42. 35062937 10.1186/s12890-022-01825-2 PMC8781590

[bibr26-13591053251369393] Quittner AL Abbott J Georgiopoulos AM , et al. (2016) International committee on mental health in cystic fibrosis: Cystic Fibrosis Foundation and European Cystic Fibrosis Society consensus statements for screening and treating depression and anxiety. Thorax 71(1): 26–34. 26452630 10.1136/thoraxjnl-2015-207488 PMC4717439

[bibr27-13591053251369393] Smith JA Flower P Larkin M (2009) Interpretative phenomenological analysis: Theory, method and research. Qualitative Research in Psychology 6(4): 346–347.

[bibr28-13591053251369393] Smith JA Flowers P Larkin M (2021) Interpretative Phenomenological Analysis: Theory, Method and Research. Sage.

[bibr29-13591053251369393] Southern KW (2025) What does the expanding CFTR modulator programme mean for people with cystic fibrosis? Lancet Respiratory Medicine 13: 195–197. 39805295 10.1016/S2213-2600(24)00427-2

[bibr30-13591053251369393] Talwalkar JS Koff JL Lee HB , et al. (2017) Cystic Fibrosis Transmembrane Regulator Modulators: Implications for the management of depression and anxiety in cystic fibrosis. Psychosomatics 58(4): 343–354. 28576305 10.1016/j.psym.2017.04.001

[bibr31-13591053251369393] White K Issac MS Kamoun C , et al. (2018) The THRIVE model: A framework and review of internal and external predictors of coping with chronic illness. Health Psychology Open 5(2): 2055102918793552. 10.1177/2055102918793552 PMC6104221 30151224

[bibr32-13591053251369393] Wise J (2023) Cystic fibrosis: what next for the ‘wonder drug’ Kaftrio? BMJ 383: 2766. 37996105 10.1136/bmj.p2766

[bibr33-13591053251369393] Zemanick ET Accurso FJ (2019) Entering the era of highly effective CFTR modulator therapy. Lancet 394(10212): 1886–1888. 31679947 10.1016/S0140-6736(19)32676-5

